# Stress Distribution Pattern in Class I Cavities Restored by Different Techniques According to the Stress Reducing Direct Composite Concept: A Finite Element Analysis

**DOI:** 10.1155/ijod/7196931

**Published:** 2025-06-04

**Authors:** Seyedeh Maryam Tavangar, Reza Tayefeh Davalloo, Farideh Darabi, Gelareh Tajziehchi

**Affiliations:** ^1^Department of Restorative Dentistry, School of Dentistry, Guilan University of Medical Sciences, Rasht, Iran; ^2^Department of Restorative Dentistry, School of Dentistry, Shahid Beheshti University of Medical Sciences, Tehran, Iran

**Keywords:** composite resins, dental stress analysis, finite element analysis, polymerization

## Abstract

**Objectives:** This study aimed to compare stress distribution in Class I cavities restored by different techniques according to the stress reducing direct composite (SRDC) concept using finite element analysis (FEA).

**Materials and Methods:** In this FEA, a model of a three-rooted maxillary molar tooth with a Class I cavity was designed using Mimics 21, Geomagic Design X, and ANSYS software programs. The cavity was restored with a conventional micro-hybrid composite in Group A, bulk-fill composite in Group B, polyethylene fiber beneath the micro-hybrid composite in Group C, and self-cure composite beneath the micro-hybrid composite in Group D. Stress distribution in tooth during resin polymerization and following the application of 600 N load was evaluated in the four groups by FEA. The maximum von Mises stress and total deformation were reported.

**Results:** Almost equal maximum stress values were detected in the enamel close to the dentinoenamel junction and particularly at the marginal ridges in all groups. The maximum stress applied to the hybrid and adhesive layers was greater in Group A, compared with other groups. More uniform stress distribution in the cavity floor was detected in Groups B and C. Stress distribution in the restorative material was less uniform in Group D. Maximum deformation was noted in Group A, followed by Groups C, B, and D.

**Conclusion:** In Class I cavities, application of polyethylene fiber beneath the micro-hybrid composite decreased the maximum stress applied to the adhesive and hybrid layers and total deformation, compared to the use of micro-hybrid composite alone; application of bulk-fill composite and self-cure composite beneath the micro-hybrid composite ranked next. Stress distribution in the cavity floor was more uniform in use of polyethylene fiber beneath the micro-hybrid composite and application of bulk-fill composite.

## 1. Introduction

The increasing demand for dental esthetics along with conservative cavity preparation and chemical bonding to tooth structure have all contributed to the growing popularity of composite restorations. However, despite the high clinical success rate of composite restorations in the posterior teeth, polymerization shrinkage is a major drawback responsible for clinical complications of composite resins.

All methacrylate-based composite resins show 1%–3% polymerization shrinkage in the process of curing [1–4]. Stress caused by polymerization shrinkage can cause composite debonding and result in postoperative tooth hypersensitivity, formation of microcracks, microleakage, and secondary caries. Weak bond of restorative material to tooth structure causes debonding at the dentin margins and subsequent microleakage, allowing the penetration of bacteria, fluids, molecules, and ions through the cavity-composite resin interface. Also, if the bond strength exceeds the polymerization stress, debonding does not occur, but the residual stress in the material causes cuspal and occlusal deflection and can result in postoperative tooth hypersensitivity and even tooth fracture in some cases [1–3, 5–7].

A number of factors such as the cavity shape, composition of composite resin, light curing technique, and composite application technique may affect the stress caused by polymerization shrinkage. Several methods have been suggested to minimize the polymerization shrinkage stress of composite resins, such as application of different intermediate layers beneath the composite resin restoration to buffer the loads. Some studies used bulk-fill composites [8–11], some used fibers [12–16], and some others used self-cure and dual-cure composite resins [17–20] for this purpose.

The concept of minimally invasive dentistry is based on caries prevention, remineralization, and conservative restoration of the teeth. Fusayama and Terachima [21] were among the first to suggest minimally invasive dentistry. The stress reducing direct composite (SRDC) technique eliminates the polymerization stress by employing a precise layering technique and light curing protocol [22]. The SRDC technique is highly important in minimally invasive dentistry because conservative preparation often results in a cavity with a high C-factor. Magne [23] described the advanced adhesive technique of “minimal stress/maximum bond” as a biomimetic technique, because this method simulates the stress/strain of natural teeth. Thus, the SRDC technique can be considered minimally invasive and biomimetic. Several factors are involved in clinical success of direct composite restoration of teeth, which are the cornerstones of the SRDC technique and include occlusal analysis, complete caries removal, precise cavity preparation, analysis of residual tooth structure, correct selection and proper application of dental bonding agents, controlling the polymerization shrinkage stress by precise layering and light curing techniques, and adjustment and standardization of occlusal forces [24].

Studies comparing different techniques for reduction of polymerization shrinkage stress by finite element analysis (FEA) are not many [25, 26]. Thus, this study aimed to compare the stress distribution pattern in Class I cavities restored by different techniques (micro-hybrid composite alone, bulk-fill composite alone, self-cure composite beneath the micro-hybrid composite, and polyethylene fiber beneath the micro-hybrid composite) according to the SRDC technique using FEA.

## 2. Materials and Methods

This FEA was conducted on four virtually simulated models of a sound human three-rooted maxillary molar tooth that had been extracted due to hopeless periodontal prognosis. The study protocol was approved by the ethics committee of Guilan University of Medical Sciences (IR.GUMS.REC.1402.313).

The external tooth surface was initially scanned by a dental scanner (LAVA, 3M ESPE, St. Paul, MN, USA) with 16 µm accuracy to create a 3D model of the tooth in STL format. The dimensions of the scan model were compared with actual dimensions of the tooth to ensure no distortion. The tooth also underwent cone-beam computed tomography and the sum of cone-beam computed tomography data and STL model (that only included the external tooth surface scan data) were transferred to Mimics and 3-Matic software programs (Materialise Mimics Innovation Suite 21.0) to create a volumetric model of the tooth.

### 2.1. Modeling in Mimics and 3-Matic Software Programs

**Figure 1:**
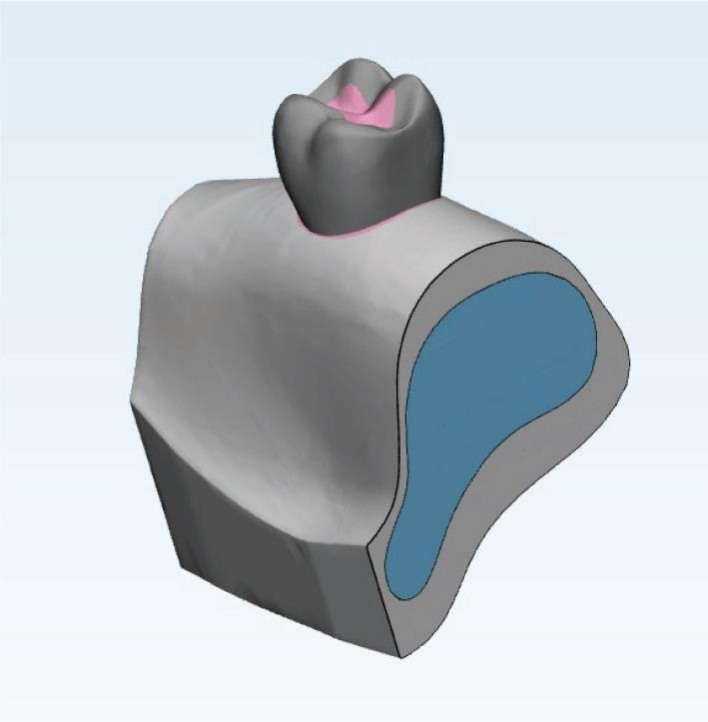
3D model of a tooth with a Class I cavity, including cortical (gray) and spongy (blue) bone.

The bone, tooth, and a Class I cavity were modeled in Mimics and 3-Matic software programs (Figure 1). The segmentation tool was used to create masks for the tooth, maxillary bone, and periodontal ligament and then their 3D models were designed by the Calculate 3D command. All parts in STL format were then exported from the aforementioned software programs.

### 2.2. Conversion of Geometries in Geomagic Software

Parts exported from Mimics and 3-Matic in STL format were converted to parts in STP format in Geomagic software (Geomagic Design X/v2020.0.3). Areas with sharp line-angles and blades were rounded by this software in order not to generate false positive stress responses in FEA.

### 2.3. FEA in ANSYS Software

**Figure 2:**
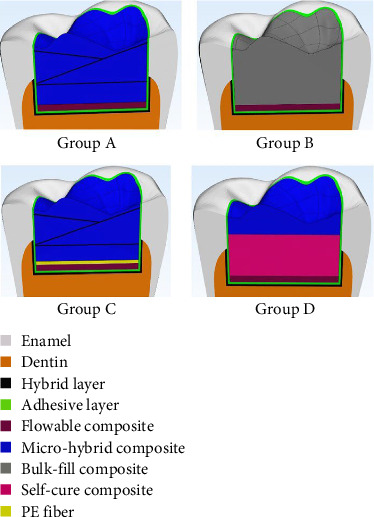
Final models of the four groups designed for FEA.

After conversion of all geometries in STP format, they were transferred to ANSYS software (ANSYS Product/v 2022 R2) for FEA. The prepared cavity was a Class I cavity with an occlusal width of 1/2 of the buccolingual width of the tooth. It had an occluso–pulpal depth of 3.5 mm [27]. All internal line-angles were rounded. The cavity was then restored by four different techniques. In all groups, adhesive and hybrid layers were included in the cavity design. The hybrid layer was designed in contact with dentin with a thickness of 5 μm and the adhesive layer was designed in the entire cavity with a thickness of 10 μm. Four groups were modeled as follows (Figure 2):

Group A (control): One layer of flowable composite (Tetric N-flow, Ivoclar, Vivadent, Schaan, Liechtenstein) with 0.5 mm thickness was applied at the cavity floor. The remaining cavity space was restored with a conventional micro-hybrid composite (Gradia Direct, GC, IL, USA) in three layers (two oblique layers and one horizontal layer).

Group B: One layer of flowable composite (Tetric N-flow, Ivoclar, Vivadent, Schaan, Liechtenstein) with 0.5 mm thickness was applied at the cavity floor. The remaining cavity space was then restored with bulk-fill composite (Tetric N-Ceram Bulk Fill; Ivoclar, Vivadent, Schann, Liechtenstein).

Group C: One layer of flowable composite (Tetric N-flow, Ivoclar, Vivadent, Schaan, Liechtenstein) with 0.5 mm thickness was applied at the cavity floor. Next, one layer of polyethylene fiber with a thickness of 0.12 mm (Ribbond, Seattle, WA, USA) was placed in the cavity. The remaining cavity space was then restored with Gradia Direct conventional micro-hybrid composite as explained for the control group.

Group D: One layer of flowable composite (Tetric N-flow, Ivoclar, Vivadent, Schaan, Liechtenstein) with 0.5 mm thickness was applied at the cavity floor. Next, one layer of self-cure composite (Briliant, Coltène/Whaledent Inc., USA) with 2 mm thickness was applied over it. Finally, one layer of Gradia Direct conventional micro-hybrid composite was applied in the cavity with 1 mm thickness with a variation range of ±0.2 mm due to the anatomical surface curvature.

### 2.4. Boundary Conditions

**Figure 3:**
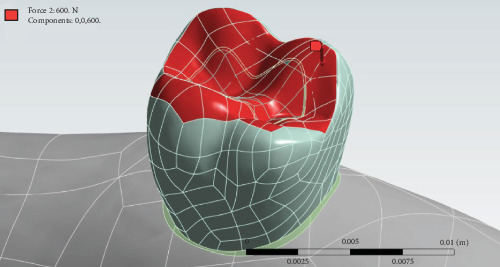
Application of a 600 N load along the tooth's longitudinal axis to simulate masticatory forces.

The attachment of enamel and dentin at the dentinoenamel junction and the attachment of dentin and pulp were both defined as cohesive, while each material preserved its own physical properties. Additionally, the hybrid layer and dentin were modeled as cohesive attachments. The adhesive attachment was defined for the adhesive layer to the hybrid layer, adhesive layer to composite, and fiber-to-composite interfaces. The composite-to-composite attachment was also modeled as cohesive. The physical properties of each material were defined in ANSYS software [11, 26–28] (Table 1). Next, 600 N load was applied to the tooth along its longitudinal axis to simulate masticatory forces (Figure 3) [26]. Also, the shrinkage force of shrinkable materials was applied in the first step as a radial contraction stress and then 600 N load was applied in the second step. The upper surface of the maxilla was fixed.

### 2.5. Meshing

Meshing was finally performed with a total number of 42,543 tetrahedral elements and 81,661 nodes.

### 2.6. Data Analysis

FEA was then performed to assess the pattern of stress distribution in tooth in the four models. The stress distribution patterns were compared among the four groups. The maximum von Mises stress values (MPa) were reported to describe the magnitude of stress applied to the tooth and composite. The von Mises shrinkage stress values were reported for the four different techniques and depicted in figures. Blue color indicated the lowest and red color indicated the highest stress value. Polymerization shrinkage stress alone and the interaction effect of polymerization shrinkage stress and occlusal force applied to the tooth were both evaluated and reported in this study.

## 3. Results

Figures 4–15 show the stress distribution pattern in the enamel, dentin, adhesive layer, hybrid layer, and restorative material. As shown in Figures 4–10, in all groups, the maximum stress accumulated in the enamel was close to the dentinoenamel junction. The stress value was greater at the center of mesial and distal marginal ridges, compared with the buccal and lingual areas.

**Figure 6:**
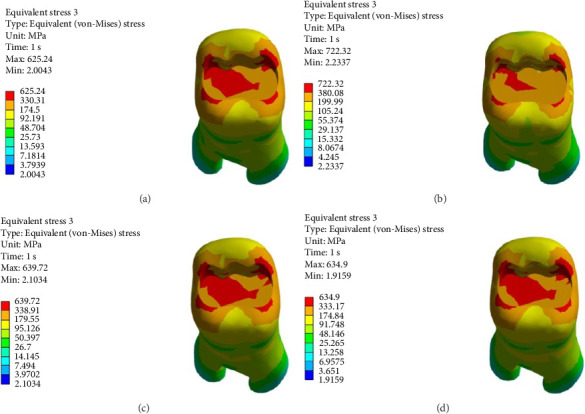
(A–D) Stress due to polymerization shrinkage applied to dentin.

**Figure 7:**
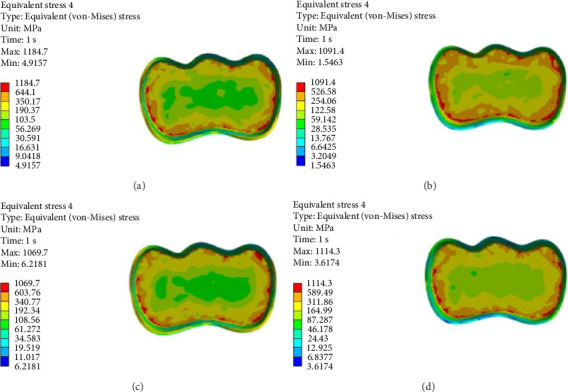
(A–D) Stress due to polymerization shrinkage applied to the adhesive layer.

**Figure 8:**
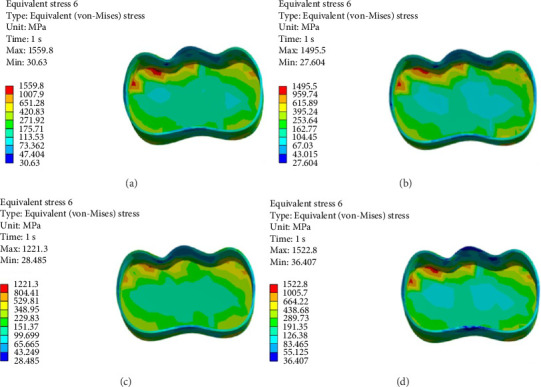
(A–D) Stress due to polymerization shrinkage applied to the hybrid layer.

**Figure 9:**
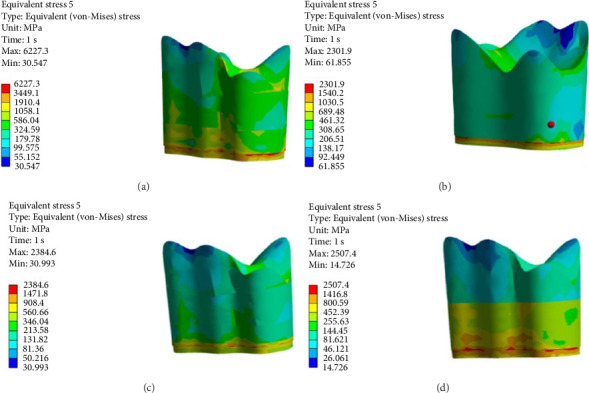
(A–D) Stress due to polymerization shrinkage applied to the restorative material.

**Figure 10:**
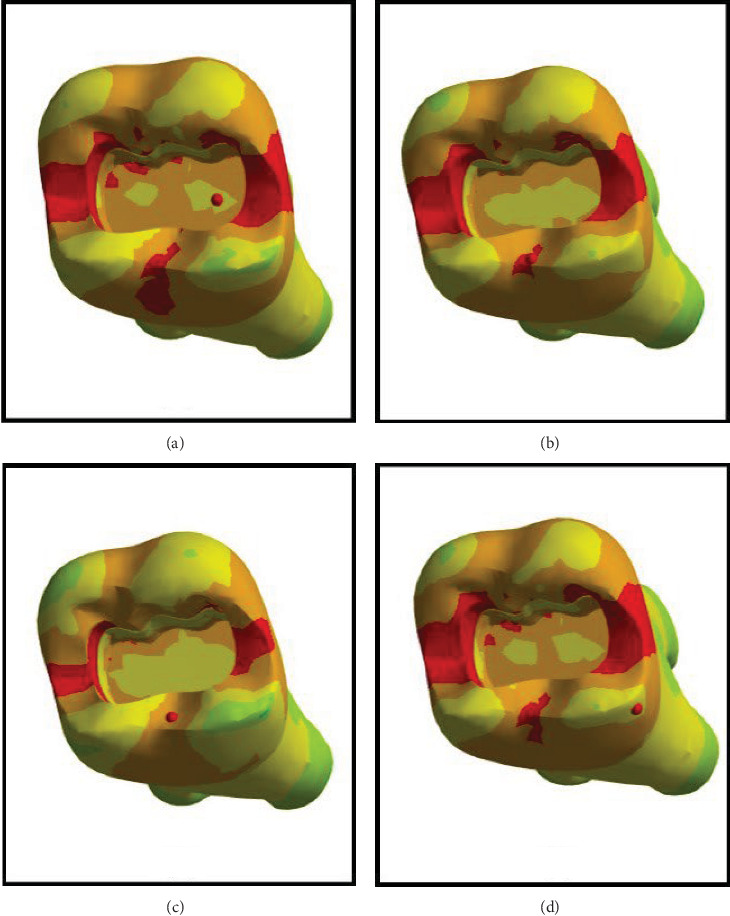
(A–D) Stress due to polymerization shrinkage and occlusal load application applied to the entire tooth.

**Figure 11:**
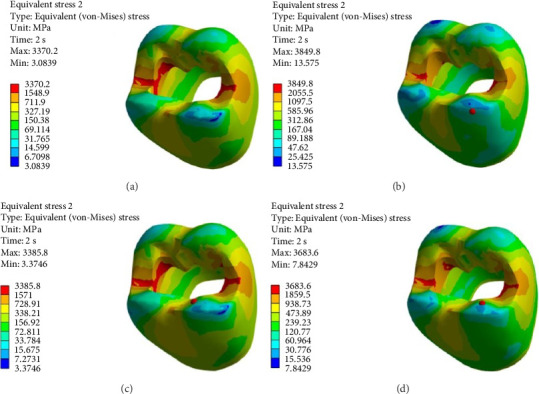
(A–D) Stress due to polymerization shrinkage and occlusal load application applied to the enamel.

**Figure 12:**
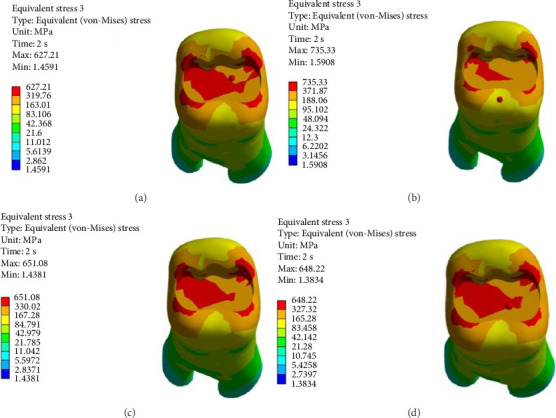
(A–D) Stress due to polymerization shrinkage and occlusal load application applied to dentin.

**Figure 13:**
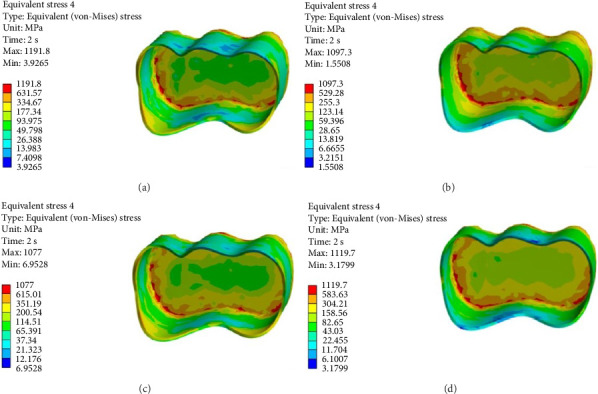
(A–D) Stress due to polymerization shrinkage and occlusal load application applied to the adhesive layer.

**Figure 14:**
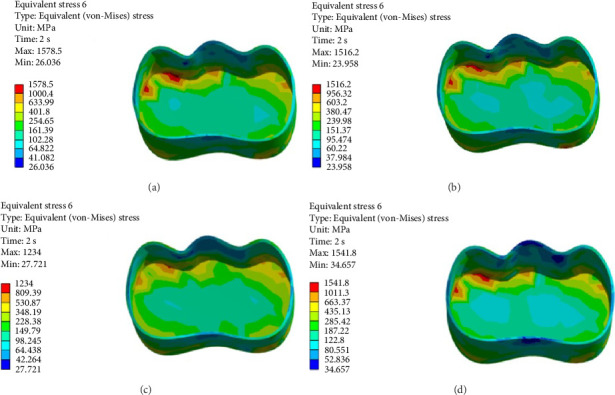
(A–D) Stress due to polymerization shrinkage and occlusal load application applied to the hybrid layer.

As shown in Figures 6–12, in all groups, the maximum stress accumulated in dentin was mainly at the corners in mesiobuccal, mesiolingual, distobuccal, and distolingual line-angles, extending to part of the cavity floor. The gingival floor area with lower stress accumulation was greater in Groups B and C. Thus, it may be stated that application of bulk-fill composite and polyethylene fiber decreased stress accumulation in the gingival floor and resulted in more uniform stress distribution. As shown in Figures 7–14, the maximum stress in the hybrid layer was at the cervical region of buccal and lingual cavity lines and close to the line-angles in all models.

**Figure 15:**
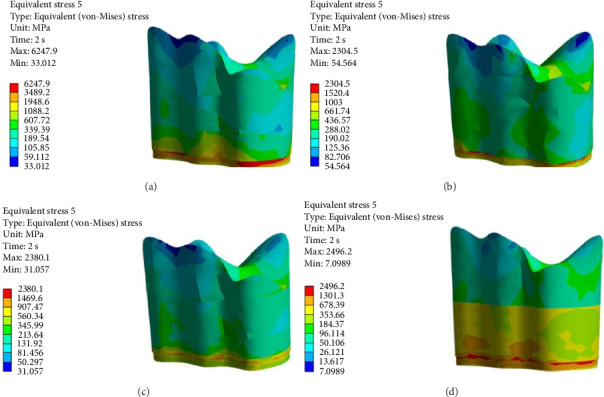
(A–D) Stress due to polymerization shrinkage and occlusal load application applied to the restorative material.

Assessment of the 3D model of restorative materials in all directions and different sections revealed maximum stress accumulation in the cervical region especially close to the flowable composite layer. In Group D, stress distribution in the restorative material complex was less uniform compared with other groups and the magnitude of stress applied to the self-cure composite was greater than that applied to the micro-hybrid composite (Figures 9 and 15).

Table 2 presents the maximum stress in the enamel, dentin, adhesive layer, hybrid layer, and restorative material as the result of polymerization shrinkage of restorative materials alone. As shown, the maximum stress in the enamel was greater when bulk-fill composite was used (Group B), compared with other groups, followed by Group D (application of self-cure composite at the cavity floor) and Group C (application of polyethylene fiber at the floor). The lowest stress accumulation in the enamel was noted in Group A (micro-hybrid composite alone).

The maximum stress in dentin was almost the same in all groups.

The maximum stress in the adhesive layer was the highest in Group A, followed by Groups D, B, and C.

The maximum stress in the hybrid layer was the highest in Group A, followed by Groups D, B, and C.

The maximum stress applied to the restorative material was the highest in Group A, followed by Groups D, C, and B.

Table 3 presents the maximum stress in the enamel, dentin, adhesive layer, hybrid layer, and restorative material as the result of polymerization shrinkage of restorative materials plus the occlusal force. As shown, application of bulk-fill composite, self-cure composite at the cavity floor beneath the micro-hybrid composite, and polyethylene fiber at the cavity floor beneath the micro-hybrid composite decreased the maximum stress applied to the adhesive and hybrid layers and increased the maximum stress applied to the enamel and dentin, compared with the use of micro-hybrid composite alone.

The total tooth deformation (in micrometers) in the four groups is presented in Table 4. As shown, deformation was the highest in Group A, followed by Groups C, B, and D.

## 4. Discussion

Evidence shows that the teeth have a complex behavior in response to masticatory forces due to the presence of different tissues in the tooth structure. Also, the masticatory forces are influenced by the muscle activity depending on the type of consumed food. The pattern of stress distribution in teeth depends on the shape of tooth and restoration, hardness, and the magnitude of applied load [28]. Enamel has a high modulus of elasticity. Dentin has a much lower modulus of elasticity than the enamel, and therefore, has greater flexibility than the enamel [26, 29].

This study compared the stress distribution pattern in Class I cavities restored by different techniques according to the SRDC technique using FEA. The results showed maximum stress accumulation in the enamel, due to its lower flexibility than other tissues. The composition of enamel and dentin is responsible for high fracture resistance of tooth during mastication [30].

Polymerization shrinkage is different from the stress caused by polymerization shrinkage. Stress is a function of the degree of polymerization shrinkage, elastic modulus, cavity shape, composition of composite resin, light curing technique, and composite application technique and does not merely depend on the magnitude of polymerization shrinkage alone [26, 31, 32]. In Class I cavities restored by the adhesive-based techniques, the shrinkage stress is internal and marginal [33].

The present study revealed that the magnitude of polymerization shrinkage stress increased in Class I cavities at the line-angles. Several studies have evaluated the role of C-factor in Class I cavities and reported results in agreement with the present findings [34–36]. However, Rodrigues et al. [37] added that increasing the C-factor did not increase stress in FEA models in their study. Obviously, several factors are involved in stress generation due to polymerization shrinkage.

The present study simulated the biomechanical response of a tooth with a restored Class I cavity to polymerization shrinkage and occlusal load application and the maximum von Mises stress value was considered as a possible cause of fracture. For this purpose, first the pattern of stress distribution caused by polymerization shrinkage of composites and then, the pattern of stress distribution due to occlusal load application was analyzed.

The hybrid layer plays a critical role in resistance against stress, particularly due to polymerization shrinkage. In the present study, the maximum stress applied to the hybrid layer was in the cervical region of buccal and lingual walls and close to the line-angles in all models. The results showed that application of bulk-fill composite, self-cure composite beneath the micro-hybrid composite, and polyethylene fiber beneath the micro-hybrid composite decreased the maximum stress applied to the hybrid layer, compared with the use of micro-hybrid composite alone. The resin bonding layer has an insignificant effect on the magnitude of stress applied to the teeth due to its low thickness of around 0.01 mm [26, 35].

Bulk-fill composite resins are highly popular for restoration of posterior teeth due to their bulk application as one increment with 4–6 mm thickness and saving time. They have improved curing properties that control for the effects of polymerization shrinkage [12, 38, 39]. Several mechanisms are used in bulk-fill composite resins to improve the depth of cure and speed of monomer conversion. In some bulk-fill composites, translucency has increased to enhance light penetration. Some other types use different photoinitiators [40].

Tetric-N-Ceram bulk-fill composite was used in the present study, which has a germanium-based photoinitiator with a curing activity higher than that of camphorquinone. Its polymerization mechanism is based on generation of two free radicals versus one free radical in types containing camphorquinone. Also, the Aessencio technology has been employed in this bulk-fill composite which results in approximation of the fraction coefficient of resin monomers and filler particles and creates a translucent structure that enhances the penetration depth of light.

The polymerization shrinkage stress is affected by the volume of fillers and elastic modulus of composite. In Tetric-N-Ceram bulk-fill composite, slower speed of curing provides a longer time to confront shrinkage forces prior to complete curing of composite resin [41]. The volumetric shrinkage of Tetric-N-Ceram bulk-fill composite is higher than that of other bulk-fill composites; however, due to its low modulus of elasticity, lower shrinkage speed results in lower magnitude of stress applied to the interface. Similar results were reported by Alavi et al. [40] and Van Ende et al. [42].

Oliveira et al. [17] evaluated the effect of different cavity restoration techniques on polymerization shrinkage stress, enamel cracks, cuspal deflection, and depth of cure in molars with mesio–occluso–distal cavities. They reported that bulk-fill composite resins resulted in lower shrinkage stress and cuspal deflection as evaluated by FEA; however, higher number of enamel cracks were seen in the bulk-fill composite group, as evaluated by transillumination. Their FEA results were in line with the present findings which showed that application of bulk-fill composite significantly decreased the stress level in the hybrid and adhesive layers, but increased the maximum stress applied to the enamel, which can result in development of enamel cracks in the clinical setting, making the area susceptible to bacterial penetration and caries development.

Martins et al. [43] reported lower stress due to polymerization shrinkage and cuspal deflection in the bulk-fill composite group compared with the incremental application of conventional composite. However, the conventional composite applied incrementally showed higher fracture resistance. Pattern of stress distribution revealed no significant difference between the bulk-fill and incremental composite groups neither in the enamel nor in dentin. In the present study, no noticeable differences in stress distribution within dentin were found across the four models, based on a comparative analysis of stress intensity and distribution patterns. However, higher stress was applied to the enamel in the bulk-fill composite group. Ausiello et al. [44] compared maximum stress in use of bulk-fill composite alone versus the bilayred application of composite (flowable composite beneath the micro-hybrid composite). They reported that the bilayered technique and application of flowable composite improved the biomechanical behavior of restoration. Similarly, one layer of flowable composite was initially applied at the cavity floor in all groups for the purpose of standardization in the present study.

Application of one layer of polyethylene fiber at the cavity floor is another suggested technique for reduction of polymerization shrinkage, which has gained recent popularity. This layer aids the first composite increment to resist the dislodging forces from the margins towards the curing light [45–47]. The Leno-weaved ultrahigh molecular weight polyethylene fibers serve as a stress absorbing layer, connect the internal tooth structures, reinforce the composite restoration in more than one direction, and improve its mechanical function [48]. Polyethylene fibers result in more efficient stress distribution in a large volume mass, due to the presence of distinct loading paths [13]. The chemical bond between the fiber and resin and the Leno-weaved nature of fibers confer resistance against crack propagation and cuspal deflection and result in redistribution of occlusal forces. Incorporation of fiber in composite resins decreases the volume of composite and subsequently lowers the volumetric shrinkage as a result of decreased volume of the organic matrix. Resultantly, polymerization shrinkage decreases and less microleakage occurs. Thus, polymerization shrinkage stress would not be high enough to cause debonding of composite from dentin and ideally, the shrinkage energy would be completely absorbed by the fiber system [47]. The bond strength of fiber to resin is highly important. It should be low enough to allow optimal function of crack cessation mechanism and energy absorption and create a safe system to protect the tooth surface against the dislodging forces. On the other hand, the fiber–resin bond strength should be high enough to tolerate forces and preserve the integrity of restoration [15]. Higher elastic modulus and lower flexural modulus of polyethylene fibers can effectively decrease stress propagation at the interface and cavity walls [49–51]. Sadr et al. [15] evaluated the effect of application of polyethylene fibers on gap formation and microtensile bond strength of deep cavities restored with bulk-fill composite by using OCT imaging and confocal laser scanning microscopy. They noticed a gap-free cavity floor in the fiber group and suggested the use of fiber as a liner and buffer under bulk-fill composite resins. They added that use of polyethylene fiber creates a fail-safe design for dental restorations. In this design, polymerization stress would not cause debonding of composite from dentin or tooth deformation and the shrinkage energy is ideally absorbed by the fiber system. Similar results were reported by Belli and Eskitascioglu [52], who used FEA to assess the pattern of stress distribution in polyethylene fibers. The present study also revealed that application of polyethylene fibers under micro-hybrid composite decreased maximum stress applied to the adhesive and hybrid layers, compared with other groups. It also decreased total deformation of tooth, compared with the use of micro-hybrid composite alone. However, Deger et al. [11] reported different results. They evaluated the effect of application of different intermediate layers under composite restorations on cuspal deformation, gap formation, and fracture strength. They reported that application of intermediate layers had no significant effect on cuspal deformation and fracture strength and application of polyethylene fiber significantly increased gap formation in Class II mesio–occluso–distal cavities.

Several studies applied fibers at the pulpal and gingival floors of Class II cavities restored with composite and reported that fibers effectively decreased polymerization shrinkage stress and microalgae [46, 53–56]. However, Dhingra et al. [57] reported that application of fibers under composite restorations had no significant effect on microleakage. Variations in the reported results in the literature may be due to differences in cavity designs and orientation of fibers.

Despite the high popularity of light-cure composite resins, self-cure composites are still used in restorative dentistry [58]. Application of one layer of self-cure composite at the cavity floor is one suggested strategy to decrease polymerization shrinkage stress, which was first suggested by Bertolotti [59] and then by Fusayama [20]. Self-cure composite resins have better marginal adaptation and lower microleakage than light-cure composite resins. They generate a different level of polymerization shrinkage stress due to two inherent properties: speed of polymerization and porosity. The main difference in internal stress between light-cure and self-cure composite resins is due to higher polymerization speed of light-cure composite resins. Lower speed of polymerization would result in better marginal adaptation of restorative material with the cavity walls. Lower polymerization speed of self-cure composite resins affects their flowability and allows more time for bonding, resulting in higher stress release [58, 60]. Also, self-cure composite resins are usually porous due to their manual fixing. Evidence shows that this porosity can decrease the progression of shrinkage stress due to the inhibitory effect of oxygen in the bubbles during the curing process and increased free surface area due to the presence of pores in the composite volume [61, 62]. Moreover, in vital teeth, it is believed that polymerization of self-cure composite occurs towards the warmer part of the tooth (center) and resultantly, composite restoration is contracted towards the tooth center [20, 59].

The present results showed that application of self-cure composite beneath the micro-hybrid composite in Class I cavities decreased the maximum stress applied to the adhesive and hybrid layers and also decreased the total deformation of tooth. Koubi et al. [63] and Atlas et al. [64] used composite resins with a self-cure component to decrease microleakage and improve marginal adaptation of Class II composite restorations and reported their positive effect. In a similar study, Bhanwal et al. [65] suggested the use of composite resins with a self-cure component as a suitable replacement for the lost dentin.

This study had some limitations. All tissues in all models were considered to be isometric, while different parts of the tooth such as dentinal tubules and enamel prisms with different orientations show different physical properties and are orthotropic in the clinical setting. Also, only one cavity design was evaluated in this study. Future studies are required on different cavity designs with different C-factors. Moreover, the effect of orientation of fibers beneath the restoration on the pattern of stress distribution should be investigated in future studies.

## 5. Conclusion

In Class I cavities, application of polyethylene fiber beneath the micro-hybrid composite decreased the maximum stress applied to the adhesive and hybrid layers and total deformation, compared to the use of micro-hybrid composite alone; application of bulk-fill composite and self-cure composite beneath the micro-hybrid composite ranked next. Stress distribution in the cavity floor was more uniform in use of polyethylene fiber beneath the micro-hybrid composite and application of bulk-fill composite.

## Figures and Tables

**Figure 4:**
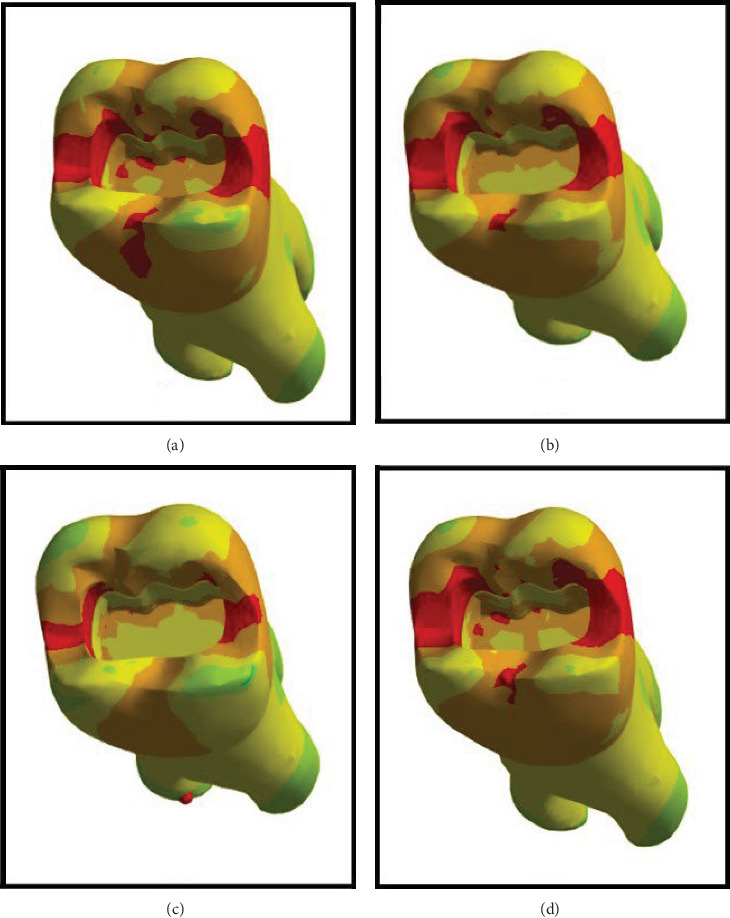
(A–D) Stress due to polymerization shrinkage applied to the entire tooth.

**Figure 5:**
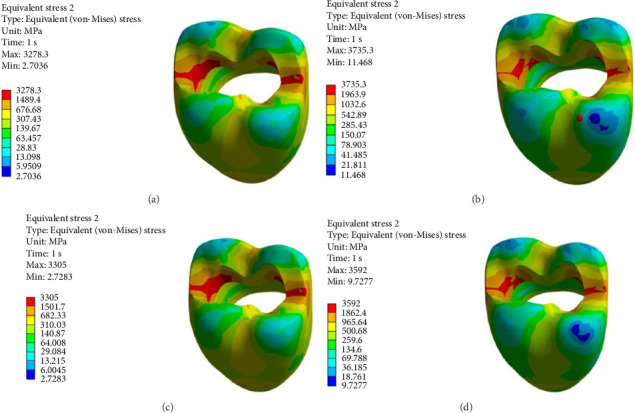
(A–D) Stress due to polymerization shrinkage applied to the enamel.

**Table 1:** Material properties.

Component	Elasticity modulus (MPa)	Poisson's ratio
Enamel	84,100	0.3
Dentin	18,600	0.3
Periodontal ligament	70	0.45
Cortical bone	1500	0.3
Spongy bone	15,000	0.3
Micro-hybrid composite	11,140	0.34
Flowable composite	5800	0.34
Bulk-fill composite	11,420	0.34
Self-cure composite	16,000	0.3
Polyethylene fiber	23,600	0.32
Adhesive layer	2600	0.28
Hybrid layer	3800	0.28

**Table 2:** Maximum stress due to polymerization shrinkage (MPa).

Component	A	B	C	D
Enamel	3278	3735	3305	3592
Dentin	625	722	639	634
Adhesive layer	1184	1091	1069	1114
Hybrid layer	1559	1495	1221	1522
Restorative material	2627	2301	2384	2507

*Note:* A: Micro-hybrid composite alone; B: bulk-fill composite; C: application of polyethylene fiber beneath the micro-hybrid composite; D: application of self-cure composite beneath the micro-hybrid composite.

**Table 3:** Maximum stress due to polymerization shrinkage and occlusal load application (MPa).

Component	A	B	C	D
Enamel	3370	3849	3385.8	3683.6
Dentin	627.2	735.3	651.08	648.2
Adhesive layer	1191.8	1097.3	1077	1119.7
Hybrid layer	1578.5	1516	1234	1541
Restorative material	2647	2304	2380	2496.2

*Note:* A: Micro-hybrid composite alone; B: bulk-fill composite; C: application of polyethylene fiber beneath the micro-hybrid composite; D: application of self-cure composite beneath the micro-hybrid composite.

**Table 4:** Total deformation (µm).

Group	A	B	C	D
Total deformation	138	129	122	121

## Data Availability

The data used to support the findings of this study will be available from the corresponding author upon request.
